# Is the Kaiser Permanente model superior in terms of clinical integration?: a comparative study of Kaiser Permanente, Northern California and the Danish healthcare system

**DOI:** 10.1186/1472-6963-10-91

**Published:** 2010-04-08

**Authors:** Martin Strandberg-Larsen, Michaela L Schiøtz, Jeremy D Silver, Anne Frølich, John S Andersen, Ilana Graetz, Mary Reed, Jim Bellows, Allan Krasnik, Thomas Rundall, John Hsu

**Affiliations:** 1 Section for Health Services Research, Department of Public Health, Faculty of Health Sciences, University of Copenhagen, Øster Farimagsgade 5, Building 10, Stairway B; 1014 Copenhagen K, Denmark; 2 Department of Biostatistics, Department of Public Health, Faculty of Health Sciences, University of Copenhagen, Øster Farimagsgade 5, Building 10, Stairway B, 1014 Copenhagen K, Denmark; 3 Copenhagen Hospital Cooperation, Bispebjerg Bakke 23, Bispebjerg Hospital, 2400 Copenhagen NV, Denmark; 4 Section of General Practice, Department of Public Health, Faculty of Health Sciences, University of Copenhagen, Øster Farimagsgade 5, Building 24; Stairway Q, 1014 Copenhagen K, Denmark; 5 Division of Research, Kaiser Permanente, 2000 Broadway; Oakland, CA 94612, USA; 6 Care Management Institute, Kaiser Permanente, One Kaiser Plaza 16th Floor, Oakland, CA 94612, USA; 7 University of California, Berkeley, School of Public Health, 50 University Hall Berkeley, CA 94720-7360, USA; 8 Mongan Institute for Health Policy, Massachusetts General Hospital, Harvard Medical School, 50 Staniford Street, Boston, MA 02114, USA; 9 Department of Health Care Policy, Harvard Medical School, 180 Longwood Avenue, Boston, MA 021115, USA

## Abstract

**Background:**

Integration of medical care across clinicians and settings could enhance the quality of care for patients. To date, there is limited data on the levels of integration in practice. Our objective was to compare primary care clinicians' perceptions of clinical integration and three sub-aspects in two healthcare systems: Kaiser Permanente, Northern California (KPNC) and the Danish healthcare system (DHS). Further, we examined the associations between specific organizational factors and clinical integration within each system.

**Methods:**

Comparable questionnaires were sent to a random sample of primary care clinicians in KPNC (n = 1103) and general practitioners in DHS (n = 700). Data were analysed using multiple logistic regression models.

**Results:**

More clinicians in KPNC perceived to be part of a clinical integrated environment than did general practitioners in the DHS (OR = 3.06, 95% CI: 2.28, 4.12). Further, more KPNC clinicians reported timeliness of information transfer (OR = 2.25, 95% CI: 1.62, 3.13), agreement on roles and responsibilities (OR = 1.79, 95% CI: 1.30, 2.47) and established coordination mechanisms in place to ensure effective handoffs (OR = 6.80, 95% CI: 4.60, 10.06). None of the considered organizational factors in the sub-country analysis explained a substantial proportion of the variation in clinical integration.

**Conclusions:**

More primary care clinicians in KPNC reported clinical integration than did general practitioners in the DHS. Focused measures of clinical integration are needed to develop the field of clinical integration and to create the scientific foundation to guide managers searching for evidence based approaches.

## Background

Within recent years the US managed care organisation Kaiser Permanente (KP) has started to influence the mindsets and policy development within many European healthcare systems [1,2]. The reason for this interest being that KP has been highlighted as a successful model of integrated and cost effective care with high quality services to their enrolees [3-5]. In the influential article by Feachem et al. the costs and performance of the United Kingdom's National Health Service (NHS) were compared with those in KP in California [3]. The authors concluded that KP provided much better value for money, largely by using only a third of the acute bed days used in the NHS. Taken at face value the benefit of the KP model was substantial. However, the claim was subsequently disputed and several serious criticisms were levelled at the methods used [6,7]. To investigate further, Ham et al. undertook a more detailed study of the KP model [4]. The findings were again in the favour of KP, with much lower hospital admission rates and overall length of stay than those of the NHS. Ham et al. indicated several factors potentially explaining the findings, including integration of funding with provision of care and integration of inpatient care with outpatient care and prevention [4]. Several commentators further indicated the importance of highly coordinated medical care and the use of clinical protocols as a driver of KP's performance [5,8-10]. However, the evidence base is far from conclusive.

KP is by definition an integrated delivery system, as an organization that unites a financing group with all providers - from hospital, clinics, and physicians through home care and long-term care facilities to pharmacies [11,12]. However, it has never been shown how this translates into delivery of integrated services at the clinical level, where it means most for the quality of care to benefit the recipients [13]. Therefore, we aim to quantify clinical integration at the primary healthcare level in KP and compare it with the level in the Danish healthcare system (DHS), a public integrated healthcare system similar to the NHS. The Danish healthcare system has been shown to be somewhat comparable to KP in terms of budget, benefits and entitlements [14]. A recent European Union Survey (PROCARE) of integrated care approaches across member states depicts Denmark and the United Kingdom as the most developed EU countries regarding implementation and testing of coordination of care strategies [15]. The Danish healthcare system is therefore a suitable comparator when attempting to benchmark the clinical integration results of Kaiser Permanente. Furthermore, we aim to examine the association between specific organizational factors and clinical integration within each system.

### Systemic conditions for clinical integration in the healthcare system settings

To set the stage for the comparative analysis we briefly present the key elements of the two healthcare systems involved (Table 1). The main focus is on the primary healthcare sector and differences between the two systems.

**Table 1:** Key elements of Kaiser Permanente, Northern California (KPNC) and the Danish healthcare system (DHS)

	KPNC	DHS
**Coverage**	Coverage according to employer enlisted or individual health plans, through Kaiser Foundation Health Plan, ranging from low coverage health plans with relatively high co-payments to plans providing extensive coverage with minimal co-payments.	Tax-based universal coverage for all residents.
	Uninsured individuals constitute 5% of total hospital admissions. 3.5% of Kaiser members are from California's Medicaid programme Medi-Cal. Medicare members can choose to obtain healthcare from Kaiser.	

**Providers**	The Kaiser Foundation Hospitals and the Permanente Medical Groups provide all clinical services.	Reliance on regional and local government for financing and delivery of healthcare services.
	The Medical Centre has a range of outpatient facilities available incl. paediatricians, internal medicine physicians, geriatricians, nurses, health educators, in-house access to advanced medical equipment, a pharmacy and an emergency department.	GPs are gatekeepers who work in private practices and are remunerated by the regions through a mix of capitation payment and fee-for-service.
	Post-hospital care is administered outside the hospitals at independent Skilled Nursing Facilities contracting with KP.	98 Municipalities are responsible for prevention and rehabilitation, home healthcare and care for the elderly.
	Physicians are paid a salary, including 5%-10% in financial incentives	Five Regions are responsible for secondary care delivered by practising specialist in private practice working under fee-for service and hospitals with physicians working for a fixed salary.

**Health Information Technology (HIT)**	The operational *KP Health Connect *allows for extensive information exchange across providers and settings. *KP Health Connect *allows for multiple patient panel management and two way patient contacts.	Widespread use of HIT but limited possibilities of information exchange across settings. To an increasing extent, GPs are using HIT for two way patient contacts. There is no common national record system.

*Source: *[1,14,19]

#### Organization, financing and primary healthcare in KPNC

KP is an integrated managed care organization founded in 1945 [16]. KP operates in nine states and Washington DC and is the largest not-for-profit managed care organization in the United States, with 8.2 million members [1]. In this study we used data from KP in Northern California (KPNC), the largest of the regional entities providing comprehensive care for 3.2 million members [1]. KPNC is a consortium of three separate but interdependent groups of entities: the Kaiser Foundation Health Plan and its regional operating organizations, Kaiser Foundation Hospitals and the Permanente Medical Groups. Kaiser Foundation Health Plan and Hospitals are integrated with legally separate physician group practices called Permanente Medical Groups. The health plan is the insurance part of the organisation, while the hospitals and medical group provide all clinical services [1,2]. To the public these hospitals and GP-type facilities are seen as one organisation, which is commonly referred to as Kaiser. The health plan and hospitals operate under state and federal not-for-profit tax status, while the medical groups operate as for-profit partnerships or professional corporations in their respective regions.

Within KP, comprehensive health services are provided, including hospital admission, sub-acute care, ambulatory and preventive care, accident and emergency, optometry, rehabilitation, and home healthcare [3]. A typical patient in need of primary care, e.g. due to a chronic condition, will, in KPNC, be treated and cared for solely in an out-patient medical centre. The medical centre will have all necessary outpatient facilities available, including paediatricians, internal medicine physicians, geriatricians, specialists, nurse practitioners, nurses, health educators, administrative personnel, a pharmacy, and an emergency department. The physicians have access to in-house laboratory facilities and other advanced medical equipment. When necessary, patients are admitted to a hospital, and subsequent care and some rehabilitation will be administered outside the hospital at a skilled nursing facility (SNIF). KP contracts with SNIFs that function as independent facilities. Integrated patient pathways are made possible by a team-based approach, multi-speciality medical centres, and information exchange across providers. The information exchange is facilitated through the operational electronic health record *KP HealthConnect*. This system also allows for multiple patient panel management and two way patient contact [16]. KPNC initiated a staggered implementation of *KP HealthConnect *in December 2004.

#### The Danish primary healthcare sector

The DHS is funded mainly through taxation and belongs to the same family of healthcare systems as those of the other Scandinavian countries and the United Kingdom [17,18]. The DHS covers all inhabitants (app. 5.4 million) and the comprehensive benefit package [19] is produced largely by public providers at the regional and local level [18]. An important exception is the General Practitioners (GP) who are self-employed, but their activities are highly regulated through agreements between their professional organization and the regional authorities. The GPs are reimbursed for their services by the regional authorities through a combination of capitation and fee-for-service. In Denmark a patient is assigned a specific GP operating in solo or multiple group practices. As of 2007, 3655 GPs were licensed to practice in Denmark. According to the Danish Association of General practitioners 37% of all GPs were organized in solo or group practices in 2007. In a solo practice the general practitioner has the sole responsibility for the patients assigned to the practice. In a group practice, a number of general practitioners share the premises and certain facilities; nevertheless, patients are still assigned to a specific GP. The remaining Danish GPs are organized in a partnership practice where two or more GPs share the responsibility for the patients as well as the economy of the practice. The GPs function as gate-keepers to the Danish healthcare system, which implies that they are the patients' first contact with the healthcare system and are expected to guide patients through the system as it relates to access to specialized care and to ensure follow-up after hospitalization. GPs have an in-house electronic health record linked to pharmacies and laboratories. Patients with a chronic condition will often need additional care provided by outpatient departments at hospitals or private practicing specialists.

## Methods

### Data on KPNC

We used data from the IMPACT2 survey. The development of this survey was based on a literature review and when possible items were derived from previously validated surveys (California Healthcare Organization Technology Adoption Survey, CMI Diabetes Survey, National Study of Physician Organizations and the Management of Chronic Illness Survey, Quality Improvement Implementation Survey II, and ULTRA Study Practice Staff Questionnaire). The IMPACT2 survey instrument was designed to measure organizational characteristics and care management practices among primary care clinicians. In autumn 2006 the survey was posted to all 1103 primary healthcare workers representing the 18 medical centers of the northern Californian region. A postal reminder followed by a telephone call was decided upon to increase the response rate. This reminder procedure was repeated up to three times. The response rate was 61% and we limited the data to the 550 respondents who indicated that they were primary care clinicians. The Kaiser Foundation Research Institute Institutional Review Board (IRB) approved the study protocol and the data were double keyed-in using Captiva Formware. Because this was a self-administered questionnaire, the IRB waived informed concent requirements, as is common practice.

### Data on DHS

We used the three-stage process stated by Fayers et al. [20] to translate the IMPACT2 survey into Danish. This process was used to improve face and content validity [20]. First, forward-backward translations were made using two independent professional translators (from English to Danish) and an expert group of health-services researchers. Inconsistencies were discussed until consensus was reached. Second, the survey underwent a peer review process among health-services researchers outside the research group, and finally we performed a field test among GPs. Special attention was given to reach conceptual and semantic equivalence [21] and ambiguous items were excluded. In Denmark the comparable profession to KPNC's primary care clinicians is GPs. In spring 2007, a Danish translation of the survey was mailed to a random sample of 700 GPs, which corresponds to app. 20% of the number of GPs in the country (n = 3655). In case of lack of response, we prepared two postal reminders. The response rate was 61% as 426 of the GPs returned the survey. Data were double keyed-in using EPIDATA http://www.epidata.dk/. Under Danish law no ethical review process was required.

### Clinical integration

Several researchers have developed models and frameworks for categorizing and assessing vertically integrated health systems [22-25]. However, few methods are validated and even fewer are validated across system settings [26-28]. In this paper we build on the theoretical framework developed by Gillies et al., in which clinical integration is identified as the most important form of integration in systems with per capita payment as in KP and DHS [18,24,29]. In this theoretical framework clinical integration is defined as:

*"The extent to which patient care services are coordinated across various functions, activities, and operating units of a given system"*. (Gillies R.R. et al. 1993)

To operationalize clinical integration we chose three core aspects of the concept: timeliness of information transfer, agreement on roles and responsibilities, and established coordination mechanisms [28]. The clinicians' perceptions of these aspects were examined by asking how often these three aspects occurred when care was transferred across clinicians (e.g. from a specialist to the primary care team). The answers were given on a 5-step Likert scale (Never - Always). We dichotomized these variables assigning 0 (never, rarely, or sometimes) or 1 (usually, always). By combining the three dichotomized variables using a summated score (0, 1, 2, 3), we gained a scale measure of clinical integration. We used Cronbach's coefficient, α_Cronbach_, to determine the internal consistency of the scale [30]. Cronbach's coefficient is useful to examine how well a set of items (or variables) measures a single unidimensional latent construct which in this case is 'clinical integration'. The observed value of α_Cronbach _for the three dichotomous response variables was 0.71 which is considered acceptable for psychometric scales [20] and an observed value >0.60 has been suggested to be sufficient for non-validated scales [31].

### Explanatory variables

The following explanatory variables were included in the analyses when comparing the two healthcare systems: system setting (DHS, KPNC), years of experience treated as a continuous variable, sex (female, male), working hours per week (full-time, KPNC at least 40 and DHS at least 37; part-time, KPNC <40 and DHS <37). In the system specific analyses, we included the aforementioned variables and in addition we included system specific covariates. In the KPNC analyses we added ethnicity (non-White, White), and implemented health information technology (HIT). To obtain the HIT variable we first considered the number of the following eight HIT features reported to be used for 81-100% of all consultations: viewing lab results, viewing the patient's current medication list, viewing patient's current drug allergies, entering orders for new prescriptions or refills, sending or receiving messages to or from other providers or staff, requesting referrals or consultations, writing free text notes, using standard note templates. This sum took values 0, 1,..., 8 and a factor variable was created as follows: limited (0-4 HIT features); some (5-6 HIT features); and extensive (7-8 HIT features). In the DHS analyses we added number of professions employed as support staff from nurses, medical secretaries, lab technicians, and dieticians (0, 1, 2, 3, 4); practice type (company practice, group practice, solo practice); number of patients used as a continuous variable with incremental steps of 100 patients. To create the HIT variable we first considered the number of the following 11 HIT features that the respondent reported to have access to in the general practice: viewing lab results, ordering new lab tests, viewing the patient's full medication list of patients, viewing the patient's medical allergies, ordering prescriptions or repeat prescriptions, communicating with health professionals outside the practice, communicating with patients, having reminders sent to patients with special health care needs, sending automatic reminders patients, booking consultations, reminders of important tests during a consultation). This sum took values 0, 1,..., 11 and a factor variable was created as follows: limited (0-5 HIT features); some (6-8 HIT features); and extensive (9-11 HIT features)

### Statistical methods

We applied a logistic regression model to estimate the association between healthcare system setting and each of the binary response variables (timeliness of information transfer; clear roles and responsibilities; established coordination mechanisms). We analyzed the ordinal scale of clinical integration using a proportional odds logistic regression model. Proportional odds logistic regression models were made for the healthcare system setting analyses as well as for each of the separate systems. Analyses were limited to respondents with complete information on all the included explanatory variables.

In the Danish setting we included an interaction term between practice size (in terms of no. of patients) and practice type. For all tests we did corrections for multiple testing, with a correction procedure based on a 5% false discovery rate (FDR). However, the presented 95% confidence intervals were not adjusted for multiple testing.

We conducted a test for non-response bias in both settings using a binominal test of proportions and a 5% significance level. In KPNC we had full information from automated registries on sex, years of experience, and ethnicity on both respondents and non-respondents. In DHS we had no information on non-respondents; accordingly, we tested whether the surveyed group of GPs was representative of their group on a national level using information on sex, regional setting, and practice type provided by the Danish General Practitioners Association. All statistical analysis was done using the statistical computer environment R [32]. The Design Package was used to fit the regression models [33].

## Results

In both system settings higher proportions of male respondents and full-time employee's respondents were found, especially in the DHS setting (Table 2). Among the respondents in the KPNC setting, we had fewer women, fewer experienced respondents, and fewer White respondents than expected. In the Danish setting the distributions on sex and practice type were similar to the distributions among all GPs in Denmark. However, we had fewer respondents in the Capital Region, more surveyed in the Central Region, and more surveyed in the Zealand Region than expected (data not shown).

**Table 2:** Frequencies of the observed variables according to the total population, the population in Kaiser Permanente, Northern California (KPNC), and the Danish healthcare system (DHS)

	Total population		KPNC		DHS	
	**N**	**%**	**N**	**%**	**N**	**%**

						

**Total population**	976	100	550	56.4	426	43.6

						

**Sex**						

Male	541	55.4	279	50.7	262	61.5

Female	430	44.1	268	48.8	162	38.0

Missing	5	0.5	3	0.5	2	0.5

						

Working hours*						

Full-time	639	*65.5*	276	50.2	363	85.2

Part-time	248	25.4	191	34.7	57	13.4

Missing	89	9.1	83	15.1	6	1.4

						

**Timeliness of information transfer**						

Yes	544	55.7	338	61.5	206	48.4

No	411	42.1	193	35.1	218	51.2

Missing	21	2.2	19	3.5	2	0.5

						

**Agreement on roles and responsibilities**						

Yes	492	*50.4*	307	55.8	185	43.4

No	456	46.7	219	39.8	237	55.6

Missing	28	2.9	24	4.4	4	0.9

						

**Established coordination mechanisms**						

Yes	330	33.8	273	49.6	57	13.4

No	616	63.1	252	45.8	364	85.4

Missing	30	3.1	25	4.5	5	1.2

						

**The combined score on clinical integration**						

0	269	27.6	132	24.0	137	32.2

1	245	25.1	87	15.8	158	37.1

2	173	17.7	87	15.8	86	20.1

3	252	25.8	215	39.1	37	8.7

Missing	37	3.8	29	5.3	8	1.9

* In KPNC full-time is at least 40 working hours per week and in DHS at least 37 working hours per week. Part-time is below 40 hours per week in KPNC and below 37 hours per week in DHS

### Comparing clinical integration across the system settings

**Figure 1:**
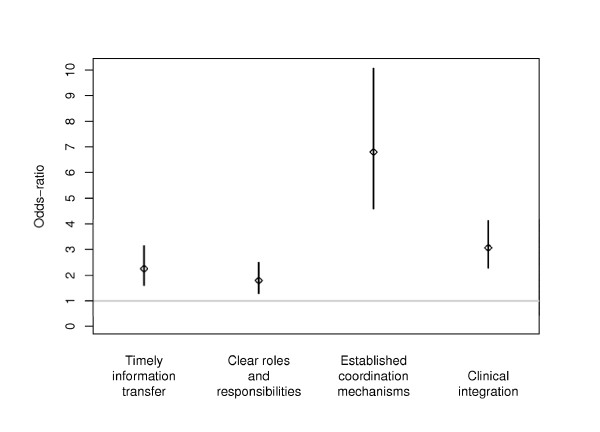
**Odds-ratios and the corresponding 95% confidence intervals for the effect of system setting (Kaiser Permanente, Northern California vs. the Danish healthcare system) on clinical integration and sub-aspects of clinical integration adjusted for differences in years of experience, sex, and working hours per week**.

More primary care clinicians in KPNC experience to be part of an environment with clinical integration and in all of the three measured sub-aspects of clinical integration than did GPs in DHS (Figure 1), even when taking into account differences in years of experience, sex, and working hours as well as corrections for multiple testing. The adjusted odds ratios of perception of clinical integration for primary care clinicians in KPNC relative to GPs in DHS was 3.06, 95% CI: 2.28, 4.12. The adjusted odds ratio of a KPNC respondent giving a positive response to the item on timeliness of information transfer was 2.25 (OR = 2.25, 95% CI: 1.62, 3.13) compared to GPs in DHS. In other words, consider the example of a male respondent in the KPNC system with 15 years' of experience and working full-time - the logistic regression predicts with a probability of 68% (95% CI: 61% - 74%) that he usually or always finds information transfer timely. The analogous probability for a Danish male GP with at 15 years' of experience and working full-time was 48% (95% CI: 42%-54%). For the other two sub-aspects of clinical integration: agreement on roles and responsibilities (OR = 1.79, 95% CI: 1.30, 2.47) and established mechanisms in place to ensure effective handoffs (OR = 6.80, 95% CI: 4.60, 10.06) system setting has a significant effect.

### System specific analysis of clinical integration

The results for the system specific analysis of clinical integration in KPNC and the DHS are presented in Table 3 and 4. As the effect of practice size on clinical integration did not differ greatly between practice types, results are shown for a regression without the interaction term. In both settings none of the explanatory variables considered, health information technology (HIT) included, could account for a substantial proportion of the system specific variation in clinical integration, especially when including corrections for multiple testing.

**Table 3:** Odds-ratios for the associations between organizational factors and clinical integration in Kaiser Permanente, Northern California

	Odds-ratio	95% CI	*p*-value	FDR *p*-value**
				

**Sex**				

Female	1.00	*Reference*		

Male	0.87	(0.59, 1.28)	0.47	0.73

				

Working hours*				

Full-time	1.00	*Reference*		

Part-time	0.98	(0.66, 1.46)	0.93	0.93

				

**Years experience**	0.99	(0.97, 1.02)	0.61	0.73

				

**Race**				

Non-White	1.00	*Reference*		

White	1.14	(0.75, 1.76)	0.54	0.73

				

**Implemented health information technology (HIT)**				

Limited	1.00	*Reference*		

Some	1.21	(0.73, 2.00)	0.46	0.73

Extensive	1.56	(0.95, 2.56)	0.08	0.49

* In KPNC full-time is at least 40 working hours per week and in DHS at least 37 working hours per week. Part-time is below 40 hours per week in KPNC and below 37 hours per week in DHS

**p-values, which are corrected for multiple testing. The correction procedure is based on a 5% false-discovery rate

**Table 4:** Odds-ratios for the associations between organizational factors and clinical integration in the Danish healthcare system

	Odds-ratio	95% CI	*p*-value	FDR *p*-value**
				

**Sex**				

Female	1.00	*Reference*		

Male	1.12	(0.74, 1.70)	0.60	0.84

				

Working hours*				

Full-time	1.00	*Reference*		

Part-time	0.86	(0.48, 1.53)	0.60	0.84

				

**Years experience**	1.01	(0.99, 1.03)	0.46	0.84

				

**Support staff**				

0 professions	1.00	*Reference*		

1 professions	1.32	(0.28, 6.19)	0.73	0.84

2 professions	0.78	(0.16, 3.71)	0.73	0.84

3 professions	0.65	(0.13, 3.29)	0.60	0.84

4 professions	1.40	(0.15, 12.76)	0.77	0.84

				

**Available health information technology (HIT)**				

Limited	1.00	*Reference*		

Some	0.68	(0.42, 1.09)	0.11	0.84

Extensive	0.92	(0.46, 1.84)	0.81	0.84

				

**Practice type**				

Company	1.00	*Reference*		

Group	1.25	(0.63, 2.49)	0.52	0.84

Solo	0.94	(0.53, 1.68)	0.84	0.84

				

Number of patients***	1.00	(1.00, 1.00)	0.48	0.84

* In KPNC full-time is at least 40 working hours per week and in DHS at least 37 working hours per week. Part-time is below 40 hours per week in KPNC and below 37 hours per week in DHS

** *p*-values, which are corrected for multiple testing. The correction procedure is based on a 5% false-discovery rate

*** Every incremental step of 1 reflects 100 patients

## Discussion

In this study we found that more KPNC primary care clinicians reported being part of a clinical integrated environment compared with an equivalent group of GPs in the DHS. It is noteworthy that a recent European Union Survey (PROCARE) of integrated care approaches across EU member states depicted Denmark and the United Kingdom as the most developed EU countries regarding implementation and testing of coordination of care strategies [15]. This study thereby contributes to the literature by bringing empirical evidence on that KPNC does have established a clinical integrated environment, and thereby supporting earlier studies that indicated the importance of highly coordinated primary care services as a driver of the performance of KPNC [4,5,8-10].

In both healthcare systems the intra-system variations in how many care clinicians reported clinical integration could not be explained by differences in sex, working hours, and years of experience. In KPNC, differences in ethnicity, and implemented health information technology (HIT) could similarly not account for intra-system variation. In the DHS the same was true for the number of professions employed as support staff, the available HIT, practice type and the number of patients assigned to a practice.

We hypothesized HIT to be a facilitating factor for clinical integration but were unable to confirm this. The findings in this study might be explained by the limited sample size and the limited variation regarding HIT in both settings. Of the 521 respondents in KPNC who provided sufficient information to calculate the 'clinical integration' variable 22% were in the 'limited' HIT category, 29% were in the 'some' HIT category and 35% were in the 'extensive' HIT category (14% had missing information on HIT). Of the 418 respondents in DHS who provided sufficient information to calculate the 'clinical integration' variable 18% were in the 'limited' HIT category, 67% were in the 'some' HIT category and 11% were in the 'extensive' HIT category (4% had missing information on HIT).

### Strengths and weaknesses

Comparative analysis is a powerful tool to highlight strengths and weaknesses in healthcare delivery systems [34,35]. When conducting comparative research one must however be aware that the specific configuration of any healthcare system depends on the historical and cultural context of health and healthcare that varies across and within countries - this complicates comparisons [36-38]. When engaging in a cross-sectional, comparative study there are therefore potential lessons to be learned but also methodological challenges and results should therefore be interpreted with care. This is the first study that quantifies clinical integration in KPNC and compares the findings to a European healthcare system. Because of the lack of valid measurement tools within the field, we consider it to be a strength of this study that we used a measurement tool on clinical integration based on a theoretical framework and that we were able to demonstrate an acceptable internal consistency of the scale (α_Cronbach _= 0.71) [26,28]. This indicates that the three items used to measure clinical integration do in fact measure a unidimensional latent structure. In the theoretical work on coordination by Alter and Hage, the authors state that perceptions of the stakeholders involved are highly important for coordination processes to take place [39] we therefore considered it to be reasonable that the measurement method used in this study uses self-reported data. Postal surveys tend to have low response rates especially among physicians. A response rate of 61% in both settings is in line with or even higher than comparable surveys [40-42], although this rate means that the possible impact of selection bias must be considered. In both settings the groups of respondents differed on some or all of the tested background variables from the non-respondents or the background population. However, adjustments for these variables had very limited impact on the estimates, and we therefore find it unlikely that the presented results are affected by selection bias to an extent that would change the estimates significantly. We have selected Danish general practitioners as the most comparable profession to primary care clinicians in KPNC. That this is a reasonable choice is a basic assumption of the present study, and we are aware that there is no perfect solution when comparing health care professionals across system settings. It was a limiting factor that the survey used to collect data in the US setting was not constructed to be used in a comparative analysis. We tried to remedy this by conducting a thorough translation process and by intensive field testing of the survey instrument in the Danish setting.

Previous studies on the association between clinical integration and organisational factors have found little evidence of widespread clinical integration [24,43-47]. Due to differences in the measurement methods, the results of these studies cannot be directly compared with this study, but it is interesting that the previous studies were conducted mainly using data from the 1990s, where the health information technology was less developed that it is today.

### Directions for future research

Additional work is needed to be carried out to obtain a fuller picture of the extent of clinical integration achieved in KPNC, DHS and other healthcare systems. The theoretical frameworks available should be further developed and tested empirically. Measurement methods should be refined and different approaches, both quantitative and qualitative, should be applied to triangulate results. Future studies should examine clinical integration based on a wider range of care professionals and should use follow-up designs that are more potent when investigating facilitating factors for clinical integration. Finally, further research needs to be conducted on the nature of integration, and on its effect on costs and benefits to healthcare delivery systems and most importantly to the patients.

## Conclusions

More primary care clinicians in KPNC reported being part of a clinical integrated environment compared to GPs in the DHS. The preferred strategy to improve clinical integration between clinicians and between settings must be based on evidence on the current level of clinical integration, intra-system variations and a clear understanding of facilitating factors and approaches to improve clinical integration. Focused measures of clinical integration are needed to develop the field of clinical integration and to create the scientific foundation to guide managers searching for evidence based approaches.

## Abbreviations

CI: confidence interval; OR: Odds Ratio; IRB: Institutional Review Board; KPNC: Kaiser Permanente, Northern California; DHS: the Danish Healthcare System; NHS: National Health Service; HIT: Health Information Technology.

## Competing interests

The authors declare that they have no competing interests.

## Authors' contributions

MSL designed the concept and conducts of the study, collected analysed and interpreted data, drafted the manuscript. MLS collected, analysed, and interpreted data and helped to draft the manuscript. JDS analyzed and interpreted data and commented on the manuscript. AF, JSA and AK assisted in study design, data analysis and interpretation, and critical revision of the manuscript for important intellectual concepts. IG and MR collected and interpreted data and commented on the manuscript. JB and TR were substantially involved in the concept and design of the data collection, and commented on the manuscript. JH analysed and interpreted data, contributed substantially to drafting the manuscript and completed critical revisions for important intellectual concepts. All authors have approved of the final submitted manuscript.

## Pre-publication history

The pre-publication history for this paper can be accessed here:

http://www.biomedcentral.com/1472-6963/10/91/prepub
